# Biomarkers in Prostate Cancer Diagnosis: From Current Knowledge to the Role of Metabolomics and Exosomes

**DOI:** 10.3390/ijms22094367

**Published:** 2021-04-22

**Authors:** Stefano Salciccia, Anna Laura Capriotti, Aldo Laganà, Stefano Fais, Mariantonia Logozzi, Ettore De Berardinis, Gian Maria Busetto, Giovanni Battista Di Pierro, Gian Piero Ricciuti, Francesco Del Giudice, Alessandro Sciarra, Peter R. Carroll, Matthew R. Cooperberg, Beatrice Sciarra, Martina Maggi

**Affiliations:** 1 Department of Urology, Sapienza Rome University, Policlinico Umberto I, 00161 Rome, Italy; stefano.salciccia@uniroma1.it (S.S.); ettore.deberardinis@uniroma1.it (E.D.B.); giovannibattista.dipierro@uniroma1.it (G.B.D.P.); gianpiero.ricciuti@uniroma1.it (G.P.R.); francesco.delgiudice@uniroma1.it (F.D.G.); martina.maggi@uniroma1.it (M.M.); 2 Department of Chemistry, Sapienza Rome University, 00161 Rome, Italy; annalaura.capriotti@uniroma1.it (A.L.C.); aldo.lagana@uniroma1.it (A.L.); sciarrajr@hotmail.com (B.S.); 3 Department of Oncology and Molecular Medicine, Istituto Superiore di Sanità, 00161 Rome, Italy; stefano.fais@iss.it (S.F.); mariantonia.logozzi@iss.it (M.L.); 4 Department of Urology and Renal Transplantation, University of Foggia, Policlinico Riuniti, 71122 Foggia, Italy; gianmaria.busetto@uniroma1.it; 5 Department of Urology, UCSF Helen Diller Comprehensive Cancer Center, University of California, San Francisco, CA 94143, USA; peter.carroll@ucsf.edu (P.R.C.); matthew.cooperberg@ucsf.edu (M.R.C.)

**Keywords:** prostate cancer, biomarkers, metabolomics, exosomes, early diagnosis

## Abstract

Early detection of prostate cancer (PC) is largely carried out using assessment of prostate-specific antigen (PSA) level; yet it cannot reliably discriminate between benign pathologies and clinically significant forms of PC. To overcome the current limitations of PSA, new urinary and serum biomarkers have been developed in recent years. Although several biomarkers have been explored in various scenarios and patient settings, to date, specific guidelines with a high level of evidence on the use of these markers are lacking. Recent advances in metabolomic, genomics, and proteomics have made new potential biomarkers available. A number of studies focused on the characterization of the specific PC metabolic phenotype using different experimental approaches has been recently reported; yet, to date, research on metabolomic application for PC has focused on a small group of metabolites that have been known to be related to the prostate gland. Exosomes are extracellular vesicles that are secreted from all mammalian cells and virtually detected in all bio-fluids, thus allowing their use as tumor biomarkers. Thanks to a general improvement of the technical equipment to analyze exosomes, we are able to obtain reliable quantitative and qualitative information useful for clinical application. Although some pilot clinical investigations have proposed potential PC biomarkers, data are still preliminary and non-conclusive.

## 1. Biomarkers in Prostate Cancer: Current Limitations

Prostate cancer (PC) is the most commonly diagnosed cancer in men, principally affecting men over 50 years old, and is the leading cause of cancer-related deaths in men [1]. Furthermore, PC and subsequent treatments have a high impact on both functional and psychological status, significantly affecting patients’ quality of life (QoL) [2]. Early detection of PC is largely carried out using assessment of prostate-specific antigen (PSA) level in blood complemented by digital rectal examination (DRE). Regrettably, PSA cannot reliably discriminate between benign prostatic hyperplasia (BPH) or prostatitis and clinically significant forms of PC, due to its limited sensitivity and specificity [3].

In 2012, the US Preventive Services Task Force (USPSTF) released a recommendation against PSA screening [4], which resulted in a reduction in the use of PSA for early detection. This strategy and recommendation led to a rise in the incidence of advanced disease and, possibly, PC cancer-related mortality after 2012 [5,6]. In 2018, the USPSTF published an updated statement suggesting that men aged 55–69 should be informed about the benefits and harms of PSA-based screening, discouraging this program for men over 70 years old [7]. A comparison of systematic and opportunistic screening suggested over-diagnosis and mortality reduction in the systematic screening group, compared to a higher over-diagnosis in the opportunistic screening regimen [8].

Over the past few years, the urgency to find an alternative approach for an early and non-invasive detection of PC, as well as for a proper discrimination between PC and several prostatic benign pathologies, has become clear. PC is a highly heterogeneous neoplasm, with many men presenting with an indolent course, while others present with a rapidly progressive disease. Due to the clinical heterogeneity of PC present in clinical practice, the analysis of the metabolic profile of PC samples is highly dispersed. Indolent PC cases with a Gleason score (GS) of 6 can display a low aggressiveness and low propensity for growth and progression; it is possible that the metabolic profile of these indolent PC cases is closer to that of BPH cases. On the contrary, clinically significant PC (csPC) cases, and in particular those with a GS of 8 or higher, often show rapid growth and progression, probably sustained by a different metabolic profile. Moreover, patients with PC usually have various extents of concurrent BPH in the transition and periurethral zones of the prostate. The determination in bio-fluids of current markers, such as PSA and its derivates, continue to be unable to properly discriminate between these two coexisting entities. The analysis of possible biomarkers, enclosed in extracellular nanovesicles released in the same bio-fluids (exosomes) rather than freely circulating, could increase their specificity and accuracy in discriminating between neoplastic and benign hyperplastic prostatic modifications [9].

## 2. Role of Different Bio-Fluids on PC Biomarkers

### 2.1. Urinary or Serum Biomarkers: Which Are Better?

In recent years, new urinary and serum biomarkers have been developed, with the goal of overcoming the current limitations of PSA, mainly represented by a low specificity, which has led to unnecessary biopsies and over-diagnosis and over-treatment of indolent PC cases [10,11,12]. Ideally, to be useful in clinical practice, a tumor biomarker should present the following characteristics: first and most importantly, it should be relatively specific for PC, and not affected by other benign conditions; second, it should be useful in all steps of the natural history of the disease (i.e., from diagnosis to follow-up after initial and subsequent therapy) and, in this context, it should be accurate in distinguishing csPC from indolent cases. Finally, biomarkers should be cost-effective, and not invasive in the method of collection [13]. In the last ten years, a better knowledge of the genetic and epigenetic mechanisms involved in PC biology has led to the availability of new urinary and plasma markers in clinical practice [14]. Although several biomarkers have been explored in various scenarios and patient settings—with the aim to identify more sensitive and specific biomarkers for detecting and monitoring PC—to date, specific guidelines with a high level of evidence on the use of these markers are lacking, mainly due to limitations inherent to both plasma and urine samples. Moreover, before widely implementing them in the different phases of patient care, there are several open questions waiting to be answered: What are the advantages of blood and urinary routes, respectively? What are the scenarios in which one biomarker is more useful than another? What is the impact—and its magnitude—of the interplay with other decision tools, such as imaging?

### 2.2. Urinary Biomarkers

Recent advances in metabolomic, genomics, and proteomics have made new potential biomarkers available, virtually in all fields of oncology. In the field of PC, these advances have led to a renewed interest in urine as a valuable biomaterial source of new markers [15]. Indeed, PC cells or substances derived from PC cells can be found in prostatic fluids—and therefore in urine samples—both directly and after prostatic massage by DRE. Therefore, urine can represent a source of prostate cells, proteins, DNA, and RNA, with the potential to serve as markers for the detection and follow-up of PC [16]. Urine has become one of the most attractive bio-fluids in clinical proteomics. Compared with other clinical biological specimens, such as blood samples, urine provides many advantages for the determination of both diagnostic and prognostic biomarkers (Table 1). 

First, urine is easy to collect—recurrently and in large quantities—without any risk or harm to the patient [17]. In addition, since it is not associated with significant proteolytic degradation and has a less complex composition compared to serum or plasma, the presence of fewer confounding elements facilitates the isolation process and thus the evaluation of biomarkers [16]. With regard to its application as a source of biomarkers for localized and early-stage PC, urine may be more appropriate than blood, as it contains markers from virtually all human tissues [16]. Moreover, urine does contain materials coming directly from the prostate gland, and it does not require crossing of blood–tissue barriers. Despite the advantages of urinary flow, only a few biomarkers are currently available and approved by regulatory authorities. The first and only FDA-approved urinary biomarker for PC is the Progensa Prostate Cancer Antigen 3 (PCA3) assay, which measures the concentration of PCA3 and PSA messenger RNAs (mRNA) levels by transcription-mediated amplification, using 2.5 mL of post-DRE urine. A PCA3 score is generated by calculating the ratio of PCA3:PSA mRNA, the latter being used as a method of normalizing for the amount of prostate material within the total volume of urine [18]. Since its introduction into clinical practice, it has shown promising results for PC detection, staging, and prognosis [19]. A recent meta-analysis showed that the sensitivity of the PCA3 test was 46.9–82.3%, and the specificity was 56.3–89% for primary diagnosis, and similar results were reported for csPC [20]. Moreover, PCA3 has proven to be useful in the context of active surveillance (AS), in which the PCA3 scores obtained at the first biopsy and during AS protocol were significantly higher in patients with Gleason grade reclassification than in those without [21]. Despite the clinical scenarios in which it has been tested, at present, PCA3 is only approved for patients with a previous negative biopsy, probably due to the fact that the definition of the best discriminating cut-off value is controversial—which has made the available studies very heterogeneous, especially in the setting of biopsy-naïve patients [22]. Indeed, several studies have highlighted the fact that PCA3 does not work well with a single threshold, showing a high NPV below a low threshold, and a high PPV above a high threshold, with a gray zone in between—which is reflective of the reality of PC biology [22,23]. These limitations might be in part overcome by combining multiple gene analysis, such as SelectMDx or Mi Prostate score [15]. SelectMDx measures post-DRE mRNA transcripts from the HOX6 and DLX1 genes in combination with other risk factors, such as age, DRE, PSA, PSA density, and family history [24]. SelectMDx has shown promising results for the initial diagnosis, with an AUC of 0.90 in the diagnosis of csPC [25]. Similar results in terms of specificity and sensibility were reported for Mi Prostate Score, which combines PCA3 with TMPRSS2-ERG and serum PSA [26]. ExoDx Prostate IntelliScore is a test that measures PCA3 and ERG RNA expression in exosomes in voided urine, without the need for a prior DRE—and thus without the need of a health-care provider to obtain the sample [27,28]. It was found to provide additional predictive accuracy above a clinical model to predict csPC, with an AUC of 0.80 [27]. In a prospective series, the addition of the gene expression model increased diagnostic performance of csPC significantly, compared to the current standard of care (AUC 0.73) [28]. 

Given the promising results of these urinary markers, to implement their use in clinical practice, some critical issues should be resolved in the future. First and most importantly, to overcome PSA limitations, future studies on new urinary biomarkers should be more focused on the diagnosis of csPC, since a biomarker that merely detects any PC will not be sufficient to improve patient care. Second, the role of DRE for the collection of urinary samples should be further investigated; yet it still remains debated, adding an element of variability among clinical studies.

### 2.3. Serum Biomarkers

Compared with urinary biomarkers, in the last years only a few blood biomarkers have been proposed and tested in PC patients, and only one is approved in clinical practice by the Food and Drug Administration (FDA). This could be partially explained by the fact that a serum biomarker should have specific characteristics, yet blood contains markers from all tissues—with a high risk of confounding factors. Moreover, blood should contain substances exclusively produced by the prostate, like PSA, and ideally not conditioned by other pathologies that can affect the prostate itself. Finally, it should be more specific than PSA for csPC. Currently, many of the new serum markers under investigation include the use of total PSA (tPSA) or free PSA (fPSA) in the analysis and interpretation of the results, leading to the issue of whether these new markers may suffer from the same limitations as PSA [14]. Ideally, optimal PC screening risk stratification requires molecular subtyping to yield information on disease biology, prognosis, and treatment benefits [29]. The prostate health index (PHI) assay and four-kallikrein (4Kscore) test have been recently developed and tested in several clinical studies, including primary diagnosis and monitoring after therapy [30]. Both tests use combinations of different serum PSA isoforms and/or related proteins to increase PC-specific sensitivity. PHI was the first FDA-approved new blood serum assay, which combines the levels of tPSA, fPSA, and p2PSA (a PC-specific fPSA isoform) [31]. Following FDA approval, several studies have focused on the comparison between the diagnostic performance of PHI and the f/tPSA ratio in different clinical settings. In the context of primary diagnosis, a large multicenter study involving more than 800 patients with PSAs between 2 and 10 ng/mL, PHI showed an AUC of 0.70 for the detection of any PC and 0.72 for csPC, highlighting its potential clinical utility for AS [31]. In this context, PHI is not recommended by scientific societies as a diagnostic tool for predicting biopsy reclassification in men under AS. However, a recent metanalysis showed a pooled sensitivity of 0.90 and specificity of 0.17 for PHI in the detection of high-grade PC [32]. The four-kallikrein (4Kscore) test is a Clinical Laboratory Improvement Amendments (CLIA) certified serum-based test that combines the levels of tPSA, fPSA, intact PSA, human kallikrein 2 (KLK2), and clinical information to obtain a risk stratification index indicating whether the patient has a csPC [33]. Similar to PHI, 4Kscore showed good accuracy for both primary diagnosis and prediction of csPC [34]. 

Table 2 summarizes data on diagnostic performance of new markers in various clinical scenarios reported from previous studies.

Despite the promising data and the numerous urinary and serum biomarkers under investigation, including FDA approved PCA3 and PHI, currently no strong recommendations by international guidelines exist [56,57]. To implement their use in clinical practice, some critical issues should be covered in the near future. Given the results of new biomarkers in reducing unnecessary biopsy and in detecting csPC, there is still a lot of uncertainty about when to use them and on which population, although a total PSA range between 2.5 and 10 ng/mL could be a valid hypothesis. Therefore, there is the need for head-to-head comparisons among new biomarkers in an attempt to understand which marker performs better for a given population. Moreover, we should take into account that multiparametric magnetic resonance imaging of the prostate (mpMRI) has radically changed the clinical practice scenario in recent years, both in patients with previous negative biopsy and in biopsy-naïve men [58,59,60,61]. Given the fact that mpMRI is the standard of care today, future research on new markers should include MRI-guided or fusion biopsies, rather than the only systematic biopsies, since the accuracy of the new markers may be higher, as reported by some authors [62,63]. In this context, combining mpMRI with biomarkers would be of particular value; yet the optimal interplay between these tools is still uncertain, as the optimal sequence and timing in order to maximize the detection of csPC while limiting the detection of indolent PC remains to be fully determined [30].

## 3. Role of Metabolomics in PC Diagnosis

Metabolomics, consisting of the exhaustive study of the entire small metabolite composition of a biological system, is considered by many as the missing link between phenotype and genotype, thus representing an essential tool in clinical study [64]. Compared with genomics or proteomics, metabolomics reflects changes in phenotype and therefore function [65]. Since metabolites represent the end-products of physiological processes, studying the metabolome may allow a better understanding of disease pathogenesis and, consequently, of the choice and mechanisms of intervention [65]. Clinical care may profit by metabolomics for several purposes, including not only the discovery of biomarkers for a specific disease, but also the metabolic differentiation of different clinical phenotypes in a cohort of patients, to stratify individuals into subgroups on which outcomes and treatments may be based. Mass spectrometry (MS) and nuclear magnetic resonance (NMR) have evolved as the most common techniques in metabolomics studies. Usually, MS approaches are combined with modern separation techniques, such as liquid chromatography (LC), gas chromatography (GC), or capillary electrophoresis (CE), depending on the physico-chemical properties of the investigated molecules. Each of these techniques brings its own advantages and limitations, which should be considered based on the analyzed samples and compounds of interest. NMR spectroscopy is a quantitative and non-destructive technique and does not require extra steps for sample preparation, such as separation or derivatization. Another strength of this technique is the high reproducibility; however, although the sensitivity of NMR spectroscopy has increased enormously, this remains a weak point for NMR compared with MS. MS-based metabolomics provides an excellent approach that can offer a combined sensitivity and selectivity platform for metabolomics research [66,67]. Compared with NMR spectroscopy, MS is superior in allowing analysis of secondary metabolites, which are present in samples in very low concentrations, such as picomole or femtomole.

Moreover, for increasing the metabolite identifications, different MS approaches, such as different ionization techniques and mass analyzer technology, can be employed. To date, two different metabolomic approaches are commonly carried out, namely targeted and untargeted metabolomics. Targeted metabolomics is focused on the quantification and identification of selected metabolites, such as those involved in a particular metabolic pathway or as the direct product of administered drugs or food intake. In targeted analysis, sample preparation plays an important role since it can reduce the ion suppression due to high abundance and interfering compounds present in complex biological sample. The MS-based metabolomics approach is the method of choice for targeted analysis compared to the NMR-based approach. Instead, untargeted approaches provide the most appropriate route to detect unexpected changes in metabolite concentrations, maximizing the number of identified metabolites. In fact, in untargeted analysis, it is possible to detect hundreds to thousands of metabolites. Moreover, no laborious sample preparation is required compared to targeted analysis. 

Untargeted approaches are usually employed when observational studies are performed, with the purpose of determining still unraveled possible biomarkers. These studies are generally performed on relatively small, but statistically significative, sets of samples. By limiting the manipulation of the samples, the broadest variety of compounds is considered. Due to the extreme complexity of biological samples, however, several minor compounds are consistently masked by high-abundance species. In a recent paper by Cerrato et al., an untargeted metabolomics study of zwitterionic and positively charged compound was set up thanks to a prior sample pretreatment step [68]. A cornerstone of any metabolomics study is the acquisition of high-quality data. This involves careful planning of experiments, analytical measurements, data processing, and statistical/chemometric analysis. Chemometrics is fundamental for obtaining reliable results after NMR and MS analyses, which provide a large amount of data. Statistical modelling, such as univariate statistical testing, multivariate regression methods (i.e., principal components analysis, partial least squares, or orthogonal projections to latent structures), cluster analysis, machine learning techniques, and non-linear methods are commonly employed for classification purposes, and selection of under—or over—expressed compounds associated with two different sets of samples [69]. Unsupervised approaches, e.g., principal component analysis, are employed for data overview for revealing outliers, groups, and trends in the groups. Conversely, supervised approaches, e.g., partial least square discriminant analysis and orthogonal projections to latent structures, are employed for building models and highlight the putative biomarkers [69]. Whenever supervised approaches are employed, particular attention on model validation must be paid to make sure that the model is not overfitted [70]. In a recent paper by Amante et al. [71], untargeted mass spectrometric data were processed by partial least square discriminant analysis in repeated double-cross validation. Untargeted approaches, therefore, counterbalance the use of small sets of samples with the need for high-performance instrumentation, multiple expertise, extensive database, and manual interpretation of the spectra. Conversely, targeted approaches are performed on generally larger patient cohorts, with a particular attention to compounds that are suspected to be linked to PC. A previous large-scale targeted study of 188 selected metabolites was performed on 777 patients, highlighting that lysophosphatidylcholines were associated with overall risk of PC [72].

PC is a disease of great interest from a metabolomics perspective for prediction, diagnosis, progression, and prognosis. A number of studies focused on the characterization of the specific PC metabolic phenotype using different experimental approaches have been recently reported (Table 3). Moreover, metabolomics approaches have been employed for determining biomarkers of PC recurrence [73]. In particular, choline phosphate has been identified as a major indicator of PC recurrence in a work by Maxeiner et al. [74]. Similarly, thioamino acid derivatives, namely cysteine, homocysteine, and cystathionine, were found to provide an increased ability in detecting recurrence over the sole clinical indices [75]. 

## 4. Can Exosomes Analysis Improve PC Biomarkers Performance?

Exosomes (Exos) are a broad and heterogeneous group of small membrane-limited extracellular vesicles (EVs) (40–180 nm in diameter) that are released from almost all mammalian cells, in both normal and pathological processes, and are thus virtually detected in all bio-fluids, including plasma and urine [86,87,88,89]. Exos are generated from the membrane invagination of endosomes and are secreted in the microenvironment after multivesicular body (MVB) fusion with the plasma membrane [86,87,90]. For this reason, Exos show specific markers obtained by budding from the endosome membranes, such as tetraspanins (CD63, CD9, and CD81), heat shock proteins (HSP70), and compounds from the Rab family, Tsg101 and Alix [86,87,88,91,92], and also other markers obtained during the process of fusion with the plasma membrane [86,87]. Moreover, Exos show a particular lipid bilayer membrane and they contain nucleic acids (i.e., DNAs, mRNAs, and microRNAs (miRNAs)) [86,87]. Cells from several organ systems (e.g., hematopoietic, gastrointestinal, nervous), as well as cancerous cells, can secrete vesicles extracellularly [91,93]. A growing body of evidence has highlighted the role of Exos as mediators of cell-to-cell communications [86,87,88,94,95,96], as well as in modulating microenvironments. Because it has been demonstrated that Exos act in the pathophysiology of different human pathologies—including cancer—they have become a promising source of disease biomarkers [86,87,97,98].

To date, nanoparticle tracking analysis (NTA), immune captured based technologies, and nanoscale flow cytometry (NFC) represent new technologies to analyze EVs, which could allow valid information—both quantitative and qualitative—for clinical application [9,99].

Different neoplasms have shown some common features, such as hypoxic conditions, low nutrient supply, and extracellular acidosis [100,101,102]. Strikingly, it has been demonstrated that, independent of the tumor histology and type, Exos are secreted in larger quantities, as well as with a smaller size, when cultured in vitro under acidic pH (6.5) compared to a physiological pH (7.4) [89]. This phenomenon is comparable to the increased plasmatic Exo levels detected in PC patients when compared to inflammatory conditions of BPH patients [99]. Given this evidence, it is possible that tumor microenvironmental acidity is responsible for the increased Exo release in cancer conditions. 

In both preclinical studies and pilot clinical trials on PC, Exo, under acidic conditions express ions transporters, such as Carbonic Anhydrase IX (CA-IX), which on Exo exerts a full enzymatic function [103]. Increased CA-IX expression has been detected in Exo from plasma of PC patients [104], suggesting that the quantification of its exosomal expression and activity may be used as a potential cancer biomarker.

The microenvironmental conditions of hypoxia and acidity may be responsible for the increased Exo release by cancer cells as well as of the increased expression of other tumor markers, including PSA [99,103].

The behavior shown by Exo under acidic pH evaluated in in vitro studies has been recently confirmed in clinical trials analyzing plasma of tumor patients with different methodologies, regardless from the tumor histotype [89,97,99]. Interestingly, a positive association between the tumor burden and the levels of plasmatic Exo has been found in pre-clinical in vivo experiments. This evidence was further confirmed by clinical trials demonstrating a dramatic decline of the plasmatic Exo levels after surgical removal of the primary tumor [105]. Overall, these data highlight the potential role of circulating Exo levels in monitoring cancer patients after surgical therapies and after/during medical treatments.

Plasmatic Exo can be characterized and quantified by an immunocapture-based ELISA (IC-ELISA) test [106], which has been recently modified and compared with other emerging technologies, such as NTA and NFC [99]. The association of these three techniques showed that acidic conditions stimulate Exo release from tumor cells [89,99]. In particular, using IC-ELISA and NFC technologies, it has been demonstrated that human PC cells secrete increased quantities of Exos expressing PSA [99]. More recently, in a prospective clinical trial comparing PC patients with both BPH and healthy controls [9], levels of Exos expressing PSA were significantly higher in plasma of PC patients, showing a significantly higher sensitivity and specificity for Exo PSA, when compared to the standard serum PSA in terms of initial diagnosis of PC [9]. In the same study, using either IC-ELISA or NFC analysis, the exosome-related measures were significantly correlated, while serum PSA and exosome PSA showed independent values [9]. Using together these two methods, sensitivity and specificity for Exo PSA in distinguishing PC from BPH were 96% and 100%, respectively, while sensitivity and specificity for IC-ELISA alone were 98% and 80%, respectively [9]. This trial was the first to demonstrate that determination of PSA inside plasmatic Exos is able to better distinguish two co-existing conditions, such as PC and BPH.

With regards to the different technologies currently available to detect plasmatic Exos in humans, several factors have made IC-ELISA a promising tool, including the following: (I) it is non-invasive; (II) it is rapid, specific, and quantitative—thus easily extendable to other conditions; (III) it requires a small quantity of sample and it is reproducible—allowing multiple readouts; (IV) it can be useful in several steps—from diagnosis to follow-up; and lastly (V), it is affordable—with reasonable costs in laboratories worldwide. Moreover, IC-ELISA analysis allows for multiple markers to be explored within the same sample and thus to quantify the expression on Exos of both known and potentially novel tumor biomarkers in the same patient.

To date, although a series of potential new PC biomarkers—including both proteins and microRNAs—have been explored in some pilot clinical trials, data are still very preliminary and non-conclusive [9,99,104,107,108,109,110,111,112] (Table 4). The implementation of IC-ELISA technology in the detection of Exo-associated mRNA may improve the quantification of plasma RNAs of tumor origin in human bio-fluids, providing more sensitive and specific analysis than quantitative real-time PCR and microarray analyses. Moreover, Exo can be enriched in mRNAs and miRNAs, which are hardly detectable in patients’ tissue, in which a high number of molecules may cover their signal.

In addition to the promising results, further research is still needed in order to validate the implementation in clinical practice of either plasmatic- or other bio-fluid-derived Exos. That would potentially allow considerable advantages for both patients and clinicians, such as avoiding or limiting unnecessary invasive procedures, and hopefully significantly reducing the public health costs.

## 5. Conclusions

The biomarker field in PC has exploded in recent years. Moreover, the available set of PC biomarkers is constantly growing (numerous novel tests, different biomaterial sources, improved analytical measurements, and statistical processing), yet only a few of them have been approved by regulatory authorities. Although the current data on PC biomarkers show great potential in aiding decision making and improved patients care, further investigation is warranted. For an optimal implementation of these tools in clinical practice, trials should be specifically designed to answer key clinical questions, and to explore whether a biomarker can actually improve PC management. Finally, combining mpMRI with biomarkers would be of particular value; yet the interplay of these tools is still uncertain, as the optimal sequence and timing remains to be determined.

## Figures and Tables

**Table 1:** Advantages and limitations of serum and urinary biomarkers.

Advantages	Critical Issues	Availability	Potential Clinical Utility
**Serum Biomarkers: PHI, 4K scores**			
Easy to perform Reproducible	High risk of confounding factors Include PSA for interpretation Include clinical variables (4Kscore) Uncertain reference range and ethnic variability (PHI)	PHI: FDA-approved 4K: CLIA-certified	Primary Diagnosis (biopsy-naïve/repeat biopsy) Diagnosis of csPC AS
**Urinary Biomarkers: PCA3, SelectMDx, MiPS, ExoDx**			
Easy to collect Large quantities Reproducible Fewer confounding elements	Need DRE (not ExoDx) Visit to a health-care provider to obtain the urine sample (not ExoDx) Difficult to collect cells derived from PC Include clinical variables (SelectMDx) Uncertain cut-off value (PCA3)	PCA3: FDA-Approved SelecMDx, MiPS, and ExoDx: CLIA-certified	Primary Diagnosis (biopsy-naïve/repeat biopsy) Diagnosis of csPC AS

Abbreviations list: PHI = Prostate Health Index; 4K = four-kallikrein; PCA3 = Prostate Cancer Antigen 3; PSA = prostate-specific antigen; DRE = digital rectal examination; PC = prostate cancer; csPC = clinically significant PC; AS = active surveillance; FDA = Food and Drug Administration; CLIA = Clinical Laboratory Improvement Amendments.

**Table 2:** Diagnostic performance of new markers in various clinical scenarios.

Biomarkers	Primary Diagnosis of PC (No. of pts, Inclusion Criteria, AUC Results)	Primary Diagnosis Repeat Biopsy (No. of pts, Inclusion Criteria, AUC Results)	Diagnosis of csPC (No. of pts, Inclusion Criteria, AUC Results)	Active Surveillance (No. of pts, Inclusion Criteria, AUC Results)
**Serum**				
PHI	No. pts 892	No. pts 95	No. pts 658	No. pts 253
	PSA 2–10 ng/mL	AUC 0.72 [35]	PSA 4–10 ng/mL	AUC 0.65 for GR [36]
	AUC 0.72 [31]		AUC 0.71 [37]	
	No. pts 658	No. pts 391	No. pts 769	
	PSA 4–10 ng/mL	PSA 2–10 ng/mL	PSA 2–10 ng/mL	
	AUC 0.71 [37]	AUC 0.78 [38]	AUC 0.72 all (0.68 initial biopsy, 0.78 repeat biopsy) [38]	
	No. pts 300	No. pts 110		
	PSA 2–10 ng/mL	PSA 2–20 ng/mL		
	AUC 0.77 [39]	AUC 0.69 [40]		
4K Score	No. pts 749	No. pts 925	No. pts 749	No. pts 718
	PSA > 3 ng/mL	PSA > 3 ng/mL	PSA > 3 ng/mL	AUC 0.78 for GR [41]
	AUC 0.69 including age and DRE [42]	AUC 0.68 including age, PSA, DRE [43]	AUC 0.78 including age and DRE [42]	
	No. pts 531		No. pts 531	
	PSA 3–15 ng/mL		PSA 3–15 ng/mL	
	AUC 0.69 including age [34]		AUC 0.71 including age [34]	
	No. pts 740		No. pts 740	
	PSA > 3 ng/mL		PSA > 3 ng/mL	
	AUC 0.83 including age, PSA, DRE [44]		AUC 0.90 including age, PSA, DRE [44]	
			No. pts 925	
			PSA > 3 ng/mL	
			AUC 0.87 including age, PSA, DRE [43]	
**Urinary**				
PCA3	No. pts 300	No. pts 48	No. pts 497	No. pts 552
	PSA 2–10 ng/mL	PSA 2.5–6.5 ng/mL	PSA > 3 ng/mL	AUC for GR 0.61 [45]
	AUC 0.73 [39]	AUC 0.79 [46]	AUC 0.53 [47]	
	No. pts 497	No. pts 470	No. pts 905	No. pts 294
	PSA > 3 ng/mL	Any PSA	PSA > 3 ng/mL	AUC for GR 0.58 [48]
	AUC 0.72 [47]	AUC 0.65 [49]	AUC 0.65 [25]	
	No. pts 578 PSA <50 ng/mL AUC 0.75, PSA 4–10 ng/mL, AUC 0.74 [50]	No. pts 103 Any PSA AUC 0.64 [51]	No. pts 138 PSA 4–20 ng/mL AUC 0.55 [52]	
SelectMDx	No. pts 52		No. pts 114	No. pts 125
	PSA > 3 ng/mL		PSA > 3 ng/mL	AUC for GR 0.70 [53]
	AUC 0.92 [54]		AUC 0.67 [55]	
			No. pts 905	
			PSA > 3 ng/mL	
			AUC 0.76 [25]	
MiPS	No. pts 1225		No. pts 1225	
	PSA > 3 ng/mL		PSA > 3 ng/mL	
	AUC 0.75 [26]		AUC 0.7 [26]	
ExoDX	No. pts 195		No. pts 195	
	PSA 2–10 ng/mL		PSA 2–10 ng/mL	
	AUC 0.73 [27]		AUC 0.80 [27]	
			No. pts 519	
			PSA 2–10 ng/mL	
			AUC 0.73 [28]	

Abbreviations list: no. pts = number of patients; PHI = Prostate Health Index; 4K = four-kallikrein; PCA3 = Prostate Cancer Antigen 3; csPC = clinically significant prostate cancer; PSA = prostate-specific antigen; AUC = area under the curve; DRE = digital rectal examination; AS = active surveillance; GR = grade reclassification; MiPS = Mi Prostate Score.

**Table 3:** Metabolomics studies focused on the analysis of bio-fluids to identify clinically relevant prostate cancer biomarkers.

Source	Experimental Approach	Sample Cohort	Main Findings	Ref
Tissue	HR-MAS combined with multivariate analysis (PLS, PLS–DA) and absolute quantification (LCModel)	no. pts = 48	Low levels of spermine and citrate are correlated with PC aggressiveness.	[76]
Prostatic fluid	1H NMR spectroscopy coupled to multiple regression analysis	no. pts = 38	Significance differences between citrate and spermine ratio in PC.	[77]
Serum	1H NMR spectroscopy coupled to multivariate analysis	no. pts = 210	Glycine, sarcosine, alanine, creatine, xanthine, and hypoxanthine were able to determine abnormal prostate (BPH + PC).	[78]
Tissue, urine, and plasma	UHPLC-MS and GC–MS	no. pts = 110	Sarcosine and N-methyl derivative of glycine were highly elevated during PC progression to metastasis.	[79]
Tissue	1H HR-MAS spectroscopy	no. pts = 20	High choline and phosphocholine levels, along with an increase in the glycolytic products lactate and alanine in PC.	[80]
Urine	UHPLC-MS/MS coupled to ROC curve analysis	no. pts = 148	Kynurenic acid was found a promising biomarker for PC detection. Sarcosine was not found as significant biomarker for the diagnosis of PC.	[81]
Serum and urine	LC–ESI–MS/MS technique and the aTRAQ reagent couple to ROC and multivariate (PLS–DA) analyses	no. pts = 89	Ethanolamine, arginine markers for PC.	[82]
Urine	ID GC/MS couple to PCA and ROC analyses	no. pts = 48	Sarcosine has no statistical difference between the PC group and in the non-PC group. Decreased urinary levels of glycine, threonine, and alanine was observed in PC group.	[83]
Urine	HPLC–TOF/MS in positive and negative polarity as well as GC–QqQ/MS couple to PCA and PLS–DA analyses	no. pts = 64	Altered levels of urinary metabolites involved in such biochemical pathways like AA, purine and glucose metabolism as well as urea and TCA cycle may be considered as potential markers of PC.	[84]
Serum	LC–MS and GC–MS	no. pts = 400	PC risk was correlated with the levels of α-ketoglutarate, thyroxine, TMAO, and erucoyl-sphingomyelin; metabolites involved in the metabolism of nucleotides, steroid hormones, and tobacco were associated with non-aggressive PC.	[85]

Abbreviations list: Ref = reference; no. pts = number of patients; PC = prostate cancer; pts = patients; HR-MAS = high resolution magic angle spinning MRS; UHPLC-MS = high throughput liquid mass spectrometry; GC–MS = gas chromatography-based mass spectrometry; NMR = nuclear magnetic resonance; ROC = receiving operating characteristics; PLS–DA = partial least squares–discriminant analysis; PCA = principal component analysis; ID GC/MS = isotope dilution gas chromatography/mass spectrometry; HPLC–TOF/M = high performance liquid chromatography coupled with time of flight mass spectrometry; GC–QqQ/MS = gas chromatography coupled with triple quadruple mass spectrometry; BPH = benign prostate hypertrophy; RP = radical prostatectomy; PSA = prostate-specific antigen; US = ultra-sound; TMAO = trimethylamine oxide; TCA = tricarboxylic acid.

**Table 4:** Summary of the clinical data obtained in prostate cancer patients using exosomes as a source of biomarkers.

Exosomal Biomarkers	Source	Isolation Method	Potential Use	Ref
PSA	Plasma	UC	Screening/Early Diagnosis	[9,99]
CA IX	Plasma	UC	Diagnosis	[104]
Survivin	Plasma	UC	Early Diagnosis	[107]
Exosomes levels	Plasma	UC	Diagnosis/Prognosis/Disease surveillance	[108]
PTEN	Plasma	UC	Diagnosis	[109]
miR-141, miR-375	Serum	FCE	Diagnosis/Stage Determination	[110]
miR-1290, miR-375	Plasma	PP	Prognosis	[111]
miR-141	Serum	PP	Diagnosis	[112]

Abbreviations list: Ref = reference; PSA = prostate-specific antigen; CA = carbonic anhydrase; PTEN = phosphatase and tensin homolog; FCE = filtration-based capture of exosomes; PP = polymeric precipitation; UC = ultracentrifugation; miR = microRNAs.

## Data Availability

Data sharing not applicable.

## Abbreviations

PC	prostate cancer
PSA	prostate-specific antigen
QoL	quality of life
DRE	digital rectal examination
BPH	benign prostatic hyperplasia
GS	Gleason score
csPC	clinically significant PC
PHI	Prostate Health Index
4K	four-kallikrein
PCA3	Prostate Cancer Antigen 3
AS	active surveillance
FDA	Food and Drug Administration
CLIA	Clinical Laboratory Improvement Amendments
mRNA	messenger RNAs
tPSA	total PSA
fPSA	free PSA
AUC	area under the curve
MiPS	Mi prostate score
mpMRI	multiparametric magnetic resonance imaging of the prostate
MS	mass spectrometry
NMR	nuclear magnetic resonance
LC	liquid chromatography
GC	gas chromatography
CE	capillary electrophoresis
HR-MAS	high resolution magic angle spinning MRS
UHPLC-MS	high throughput liquid mass spectrometry
GC–MS	gas chromatography-based mass spectrometry
ROC	receiving operating characteristics
PLS–DA	partial least squares—discriminant analysis
PCA	principal component analysis
ID GC/MS	isotope dilution gas chromatography/mass spectrometry
HPLC–TOF/M	high performance liquid chromatography coupled with time of flight mass spectrometry
GC–QqQ/MS	gas chromatography coupled with triple quadruple mass spectrometry
RP	radical prostatectomy
US	ultra-sound
TMAO	trimethylamine oxide
TCA	tricarboxylic acid
Exo	exosomes
EVs	extracellular vesicles
MVBs	multivesicular bodies
miRNAs	microRNAs
NTA	nanoparticle tracking analysis
NFC	nanoscale flow cytometry
CA-IX	carbonic anhydrase IX
IC-ELISA	immunocapture-based ELISA
PTEN	phosphatase and tensin homolog
FCE	filtration-based capture of exosomes
PP	polymeric precipitation
UC	ultracentrifugation
